# Supplementary material for: Genome-Wide Identification and Molecular Evolutionary History of the Whirly Family Genes in Brassica napus
Source: Plants (Basel). 2024 Aug 13;13(16):2243. doi: 10.3390/plants13162243 (PMC11359715; doi:10.3390/plants13162243)
Supplement: Supplementary file 1 [file plants-13-02243-s001.zip › Supplementary Figure S1.pdf]

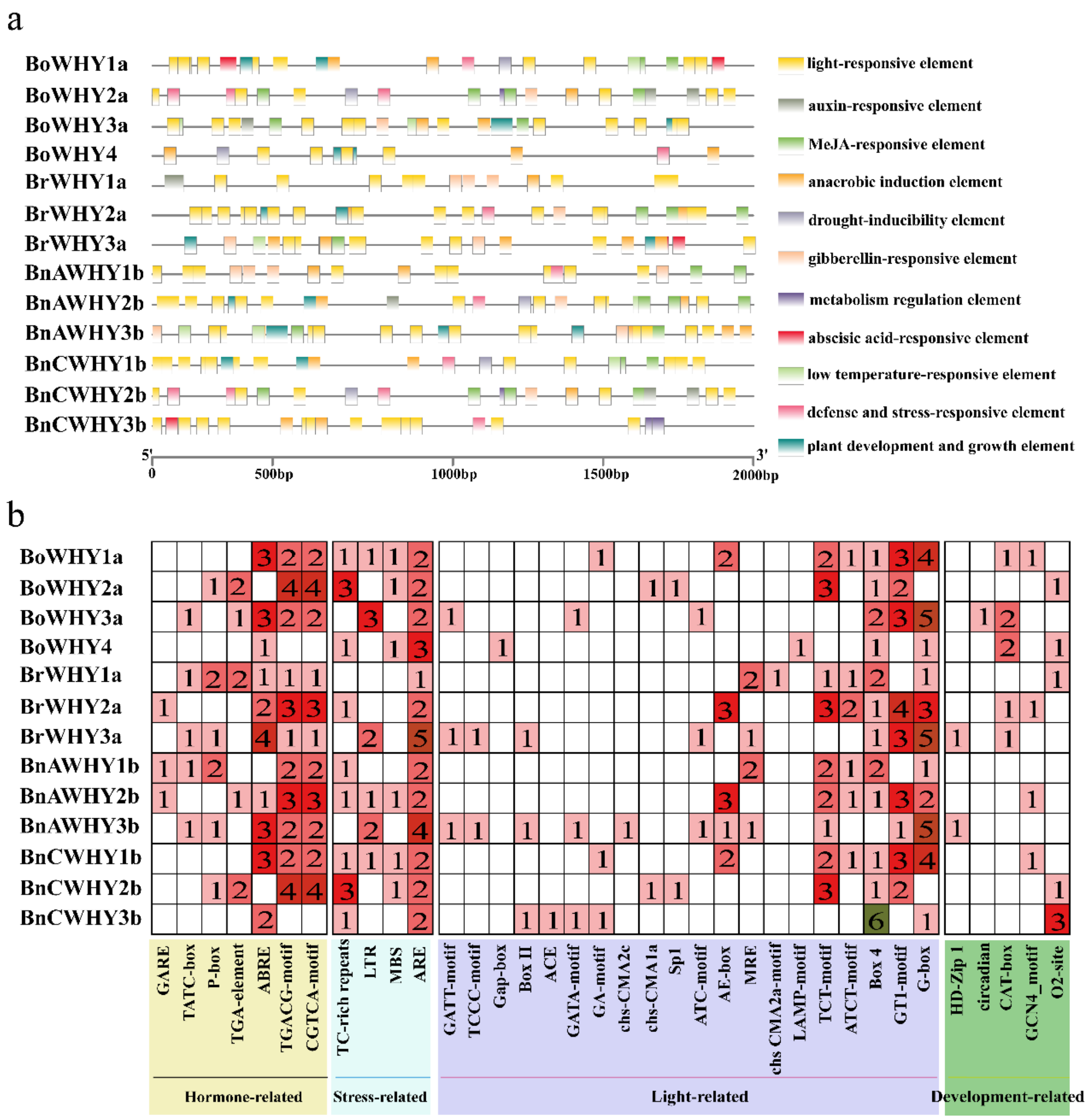

Supplementary Figure S1. Analysis of cis-acting elements in the promoter region of the *Whirly* gene family. (a) Different colored boxes represent different types of cis-acting elements in the promoter region, with the scale line below indicating the base length (bp) of the promoter region. (b) The numbers and shades of color inside the heatmap boxes indicate the quantity of cis-acting elements.
